# Chronotherapy for morning blood pressure surge in hypertensive patients: a systematic review and meta-analysis

**DOI:** 10.1186/s12872-021-02081-8

**Published:** 2021-06-04

**Authors:** Ziyan Xie, Jiahao Zhang, Chenyu Wang, Xiaowei Yan

**Affiliations:** Department of Cardiology, Peking Union Medical College Hospital, Peking Union Medical College & Chinese Academy of Medical Sciences, No.1 ShuaiFuYuan, Beijing, 100730 China

**Keywords:** Morning blood pressure surge, Circadian rhythm, Chronotherapy, Administration time, Hypertension

## Abstract

**Background:**

The morning blood pressure surge (MBPS) is related to an exaggerated risk of cardiovascular diseases and mortality. With increasing attention on circadian change in blood pressure and extensive use of ambulatory blood pressure monitoring (ABPM), chronotherapy that administration of medication according to biological rhythm, is reported to improve cardiovascular outcomes. The aim of this study is to evaluate the influence of chronotherapy of antihypertensive drugs upon MBPS in hypertensive patients.

**Methods:**

A search strategy was applied in Ovid MEDLINE, EMBASE, Cochrane (Wiley) CENTRAL Register of Controlled Trials, Cochrane Database of Systematic Reviews, and the Chinese Biomedical literature database. No language and date restrictions. Randomized controlled trials (RCT) assessing the efficacy of evening and morning administration of the same medications in adult patients with primary hypertension were included.

**Results:**

A total of ten trials, comprising 1724 participants with a mean age of 61 and 51% female, were included in this study. Combined analysis observed significant reduction of MBPS (− 5.30 mmHg, 95% CI − 8.80 to − 1.80), night-time SBP (− 2.29 mmHg, 95% CI − 4.43 to − 0.15), night-time DBP (− 1.63 mmHg, 95 %CI − 3.23 to − 0.04) and increase in night blood pressure dipping (3.23%, 95% CI 5.37 to 1.10) in evening dosage compared with traditional morning dosage of blood pressure-lowering drugs. No significant difference was found in the incidence of overall adverse effects (RR 0.65, 95% CI 0.30 to 1.41) and withdrawal due to adverse effects (RR 0.95, 95% CI 0.53 to 1.71).

**Conclusions:**

Our study suggested that evening administration of antihypertensive medications exerted better blood pressure-lowering effect on MBPS compared with conventional morning dosage. Safety assessment also indicated that the evening regimen did not increase the risk of adverse events. However, endpoint studies need to be carried out to confirm the significance and feasibility of this treatment regimen in clinical practice.

**Supplementary Information:**

The online version contains supplementary material available at 10.1186/s12872-021-02081-8.

## Background

The circadian rhythm plays a critical role in multiple neurohormonal processes, thus modulating the cardiovascular system [1, 2]. The circadian changes in blood pressure have received increasing interest. Additionally, with the introduction of ambulatory blood pressure monitoring (ABPM) into regular hypertension management, day-night patterns of blood pressure can be observed [3]. Early morning is the time of the highest incidence of cardiovascular events during the day [4]. Accumulating evidence demonstrated that an exaggerated morning blood pressure surge (MBPS) is closely related to the increased risk of cardiovascular diseases and all-cause mortality [5–8]. Besides, systematic review also indicated that when using a continuous variable to test correlations, a 10 mm Hg increase in MBPS was related to high risk of stroke [9]. Thus, the control of MBPS is clinically relevant and important. In addition, night blood pressure dipping, an index of blood pressure decline, has also been reported as an important prognostic marker for cardiovascular events and morbidity in hypertensives [10, 11].

Chronotherapy is the adaption of medication to biological rhythm, to achieve maximal effectiveness by altering the time of drug administration [12], which could influence the pharmacokinetic properties of antihypertensive medications. Multiple works have evaluated the administration-time effects in hypertension therapy. Hermida et al. revealed that administration of at least one prescribed antihypertensive medications in hypertensives at bedtime, compared to upon wakening, significantly improved blood pressure control (especially bedtime blood pressure) and remarkably diminished the occurrence of major cardiovascular events, such as stroke, myocardial infarction, death, etc. [13]. A Cochrane systemic review showed that bedtime ingestion of antihypertensive drugs was more effective to decrease 24-h blood pressure without additional adverse effects than morning regimen [14].

Some studies documented that bedtime ingestion of blood pressure-lowering agents had a more efficient antihypertensive effect during night-time and at the early morning period [15–17]. Most studies working on the administration time of antihypertensive medications were small-scaled and single-centered. And the bedtime administration has not been recommended in guidelines. Whether the bedtime administration of antihypertensive medications has a more significant effect on lowering MBPS than conventional morning administration has not been reported in any systematic review and meta-analysis. Therefore, this systematic review was conducted to investigate the efficacy of chronotherapy of once-daily antihypertensive drugs on reducing MBPS, systolic and diastolic blood pressure, and adverse effects in hypertensive patients, thus providing more evidence to the bedtime regimen.

## Methods

The protocol for this systematic review and meta-analysis was registered in PROSPERO (CRD42020180166).

### Search strategy

The searching databases included: Ovid MEDLINE, EMBASE, Cochrane (Wiley) CENTRAL Register of Controlled Trials and Cochrane Database of Systematic Reviews, the Chinese Biomedical literature database up to April 2020. Besides, we also tracked completed clinical trials that met our inclusion criteria on ClinicalTrials.gov. The major search terms included: “hypertension”, ‘‘morning surge”, “morning blood pressure surge”, “MBPS”, “night* decline”, “Chronotherapy”, “morning OR day OR am OR diurnal OR daytime OR awake”, “evening OR bedtime OR night OR nocturnal OR pm” (shown in Additional file 1: Table 1). In addition to databases, studies in references lists of relevant articles that met our criteria were also hand-searched and screened as a significant supplement. No language and date restrictions were applied to avoid missing any related investigations.

**Table 1:** Characteristics of included studies

Study	Year	Country	Study size	follow-up period (week)	Mean age (mean ± SD if available)	Sex N (%) females	Hypertensive status	Medication	Compliance evaluation	Outcome
Hermida et al.	2009	Spain	238	8	53.3 ± 11.4	130 (55%)	Grade 1 or 2 essential hypertension^a^	Nifedipine-GITS (CCB)	Tablet counts and interviews	①②③④⑤⑥⑦⑨
Hoshino et al.	2010	Japan	31	32	69 ± 11	19 (61%)	Essential hypertension^b^	Olmesartan + Amlodipine (CCB + ARB)	Not mention	①②③④⑤⑥⑦
Acelajado et al.	2012	USA	38	8	51.7 ± 11.6	17 (46%)	Grade 1 or 2 essential hypertension^a^	Nebivolol (β-blocker)	Professional instruction	①
Peng et al.	2013	China	54	8	58.3 ± 10.7	26 (48%)	Grade 1 or 2 essential hypertension^a^ and 24 h mean ambulatory blood pressure more than more than 130 / 80 mm Hg	Telmisartan + Amlodipine (CCB + ARB)	Not mention	②③④⑤⑥⑦
Zhang et al.	2014	China	156	8	56.3 ± 6.1	92 (59%)	Essential hypertension^b^	Amlodipine + Losartan (CCB + ARB)	Not mention	①②③④⑤⑥⑦
Dion et al.	2015	Germany, Spain, France, Italy and the Netherlands	639	12	61.6 ± 10.6	281 (44%)	Grade 1 or 2 essential hypertension^a^ and 24 h mean ambulatory BP (maBP) more than 130/80 mmHg	Valsartan (ARB)	Professional instruction	①②③④⑤⑥⑦⑧⑨⑩
Lai et al.	2015	China	120	2	60.6 ± 5.3	55 (46%)	Essential hypertension^b^	Losartan (ARB)	Not mention	①
Qiao et al.	2015	China	108	4	64.7 ± 8.3	62 (57%)	Essential hypertension^b^	Candesartan (ARB)	Not mention	①④⑤⑥⑦
Zhao et al.	2015	China	244	48	74.5 ± 9.1	104 (43%)	Essential hypertension^b^	Nifedipine-GITS (CCB)	Interviews every two weeks	①②③④⑤⑥⑦⑧
Li et al.	2016	China	96	12	65.1 ± 9.4	49 (51%)	Essential hypertension^b^	Enalapril (ACEI)	Not mention	①②③④⑤⑥⑦

① MBPS, ② 24 h SBP, ③ 24 h DBP, ④ daytime SBP, ⑤ daytime DBP, ⑥ night-time SBP, ⑦ night-time DBP, ⑧ overall adverse effects, ⑨ withdrawals due to adverse effects, ⑩ serious adverse effects

MBPS, morning blood pressure surge; SBP, systolic blood pressure; DBP, diastolic blood pressure; CCB, calcium channel blockers;β-blockers, beta-antagonists; ARB, angiotensin II receptor blockers; ACEI, angiotensin converting enzyme inhibitors; GITS, gastrointestinal therapeutic system formulation

^a^According to European Society of Hypertension–European Society of Cardiology guidelines: systolic blood pressure 140–179 mmHg and/or diastolic blood pressure 90–109 mmHg

^b^According to Chinese guidelines for the management of hypertension: Systolic blood pressure more than 140 mmHg and/or diastolic blood pressure more than 90 mmHg

### Inclusion and exclusion criteria

Study selection was performed independently by two reviewers (Z.X. and J.Z.) by viewing the titles and the abstracts of the search strategies. The disagreements were judged by a third reviewer who was blinded to the first two reviewers’ decisions. The selection process was repeated twice by each reviewer. Studies meeting the inclusion criteria were checked in detail (full text).

Only randomized control trials (RCT) were included to assess the effects of chronotherapy. Randomized cross-over trials that only had two treatment periods (two interventions) were also considered. Adults (more than 18) with primary hypertension, which is defined as systolic and/or diastolic blood pressure levels more than 140/90 mmHg were included. Secondary hypertension, alternating shift workers, and severe cardiac insufficiency (NYHA III-IV) were the exclusion criteria. Studies reporting one-drug therapy or combined therapy (two or more drugs) with the antihypertensive drug(s) administered once daily at bedtime when the control group was the same drug(s) at the same dose(s) once a day upon awakening were included. Antihypertensive drugs comprised diuretics, adrenergic beta-antagonists (β-blockers), alpha-antagonists, calcium channel blockers (CCB),vasodilator agents and renin-angiotensin system inhibitors (RASI, including angiotensin II receptor blockers and angiotensin-converting enzyme inhibitors). Administration time in the evening was from 18:00 to 24:00, while in the morning was from 6:00 to 12:00.

Included studies must clearly define and measure MBPS. Both continuous and categorical variables were included. Definitions of MBPS must belong to any one of the following: (1) the sleep-trough surge, calculated by the mean value of morning blood pressure within 2 h after waking minus the lowest night-time blood pressure; (2) the prewaking surge, the mean blood pressure within 2 h after waking to subtract the mean blood pressure within 2 h before waking; (3) the rising blood pressure surge, morning blood pressure reading upon rising minus blood pressure reading in the lying position 30 min before rising. The measurement of night dipping is the percentage of reduction in mean nighttime blood pressure relative to mean daytime blood pressure.

### Outcomes

The primary outcome was the change in MBPS from baseline to the end of treatment (or the value of MBPS at the end of treatment if the baseline is comparable) when it was calculated as a continuous variable; or the ratio of patients whose MBPS exceeded the settled threshold after treatment when MBPS was defined as a categorical variable.

The secondary outcomes contained night blood pressure dipping, 24-h mean systolic blood pressure (SBP) and diastolic blood pressure (DBP), daytime mean SBP and DBP (measured by ABPM from the time patients wake up in the morning to the time they fall asleep in the evening or from 6–8:00 to 22–24:00), night-time SBP and DBP (measured by ABMP from the time patients fall asleep in the evening to the time they wake up in the morning or from 22–24:00 to 6–8:00). Safety outcomes include overall adverse effects and withdrawals due to adverse effects during treatment. Adverse effects can be any unplanned and unfavorable symptom, or disease temporally associated with the use of medicine. Withdrawals due to adverse effects are reported as events leading to permanent trial discontinuation.

### Data extraction and management

Data were extracted by two independent reviewers (Z.X. and J.Z.) based on a standard form. Necessary information of studies was extracted, such as patients’ demographics (age, sex, region, race, hypertensive status, medication history), study methods (patients’ recruitment, randomization, crossover, treatment duration), interventions (drugs, dose, administration time), outcomes (definition and measurement of MBPS, night blood pressure dipping, 24-h mean SBP/DBP, daytime mean SBP/DBP, night-time SBP/DBP) and safety endpoints (headache, nasopharyngitis, edema, bronchitis, pain and withdrawals due to adverse effects). The numeric data (e.g., blood pressure) were collected from text and tables. Data in graphs were not extracted due to a possible measurement error. All the studies were double coded by the two reviewers. If there were missing data, we attempted to contact the authors to provide the missing information. Regarding missing data for the standard deviation of the change in MBPS, imputation was conducted based on other similar trials.

### Risk of bias assessment

Evaluation of the risk of bias in all included trials was executed by two independent reviewers (Z.X. and J.Z.) based on the guide of the Cochrane risk of bias tool. Characteristics of assessment included random sequence generation, allocation concealment, blinding, incomplete outcome data, selective reporting, and other bias (e.g., conflict of interest). The assessment was done at the study level.

### Data analysis

All data analysis and synthesis in the meta-analysis was conducted by RevMan 5.4 (RevMan 5.4; Cochrane Collaboration, Oxford, UK). Comparisons of continuous MBPS changes, 24-h, daytime and night-time SBP and DBP between groups were presented as mean ± standard deviation (SD) with corresponding 95% confidence intervals. These data were entered using a generic inverse variance. Categorical MBPS, overall adverse effects, and withdrawals were presented as relative risk ratios (RR). The outcomes were combined using a fixed- effect model (I^2^ ≤ 25% ) and a random-effect model (I^2^ > 25%).

### Subgroup analysis

Subgroup analysis were conducted by classifying the trials into those using different MBPS definitions, and those using antihypertensive drugs from different classes. Subgroup analysis can help locate the sources of heterogeneity and assess the effectiveness of different kinds of medicines.

## Results

### Search results

**Fig. 1:**
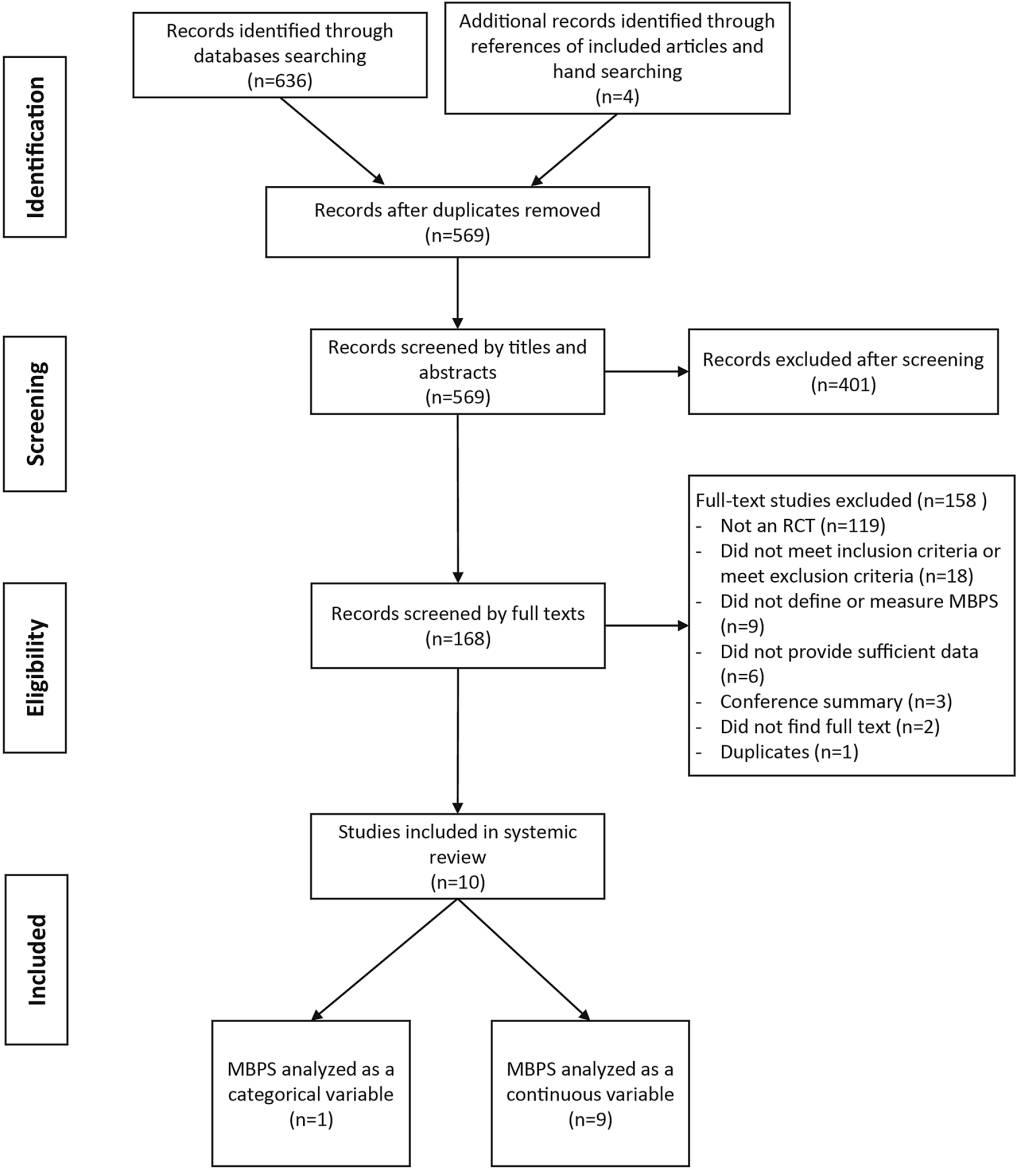
Search flow diagram for the process of search

636 records were detected after database searching, and 4 additional articles were hand-searched in reference lists of published papers. After the removal of duplicates, 569 articles were viewed by titles and abstracts by two independent reviewers (Z.X. and J.Z.), 401 of which were excluded, and the remaining 168 references entered full-text screening for intensive evaluation. Eventually, 10 articles (10 trials) were considered for analysis and involved in our study. The reasons for excluding 158 references are shown in Fig. 1.

### Description of included studies

Included studies were 8 parallel-designed RCTs and 2 crossover-designed RCT [18] with 1724 participants in 8 different countries. The key characteristics of the trials are listed in Table 1. The study size ranged from 31 to 639. The mean age of patients was 61 years, and females accounted for 43–61%. The duration of follow-up periods differed from studies (ranged from 2 to 48 weeks). Eight studies described ethnicity. One trial was conducted in the USA, with 38 participants (2%), half of which were African American [18]. One trial (37%) was a multi-center study conducted in 94 centers in five countries with nearly 99% white [19]. Six studies with 778 (45%) participants were recruited from China [20–25]. The remaining two trials without reporting race were organized in Europe (14%) and Japan (2%) [26]. The definitions and measurements of MBPS were listed in Additional file 1: Table 2. Peng et al. analyzed MBPS as a categorical variable [25], and the study of Zhang et al. contained both continuous and categorical variables [20]. Among all the trials, seven studies presented secondary outcomes [19–23, 25, 26].

### Risk of bias in the included studies

The overall assessment of the risk of bias in the included studies are shown in Additional 1: Table 3. One study, as a crossover RCT, only the first period data were available [18]. One study had the risk of baseline imbalance [22]; six studies provided insufficient information about other bias [19, 21, 23–25]. Good random sequence generation, concealed allocation and blinding were ascertained in all the studies. And none of the trials had attrition bias.

### Effects of interventions

#### Changes in MBPS

**Fig. 2:**
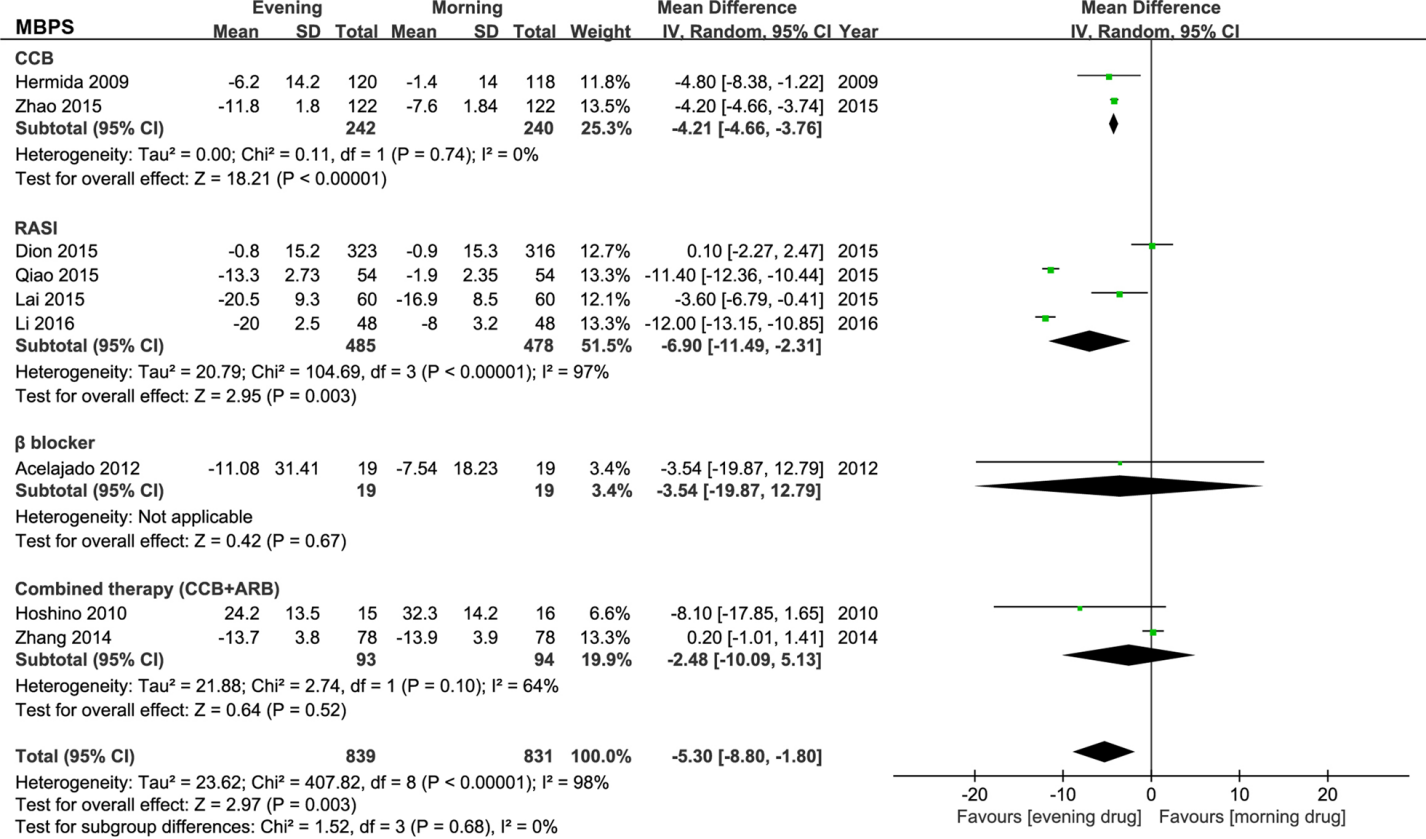
Forest plot: meta-analysis of antihypertensive drugs and MBPS. MBPS, morning blood pressure surge; CCB, calcium channel blockers; RASI, renin-angiotensin system inhibitors; β-blockers, beta-antagonists; ARB, angiotensin II receptor blockers

The most common definition of MBPS (sleep-trough surge) were compared in included studies (Fig. 2). The overall analysis showed that the evening administration significantly decreased MBPS by 5.30 mmHg (95% CI − 8.80 to − 1.80). But a significant heterogeneity (I^2^ = 98%) was detected.

The subgroup analysis observed significant differences in the evening dosage regimen of CCB and RASI compared with morning administration. The evening administration of CCB lowered MBPS by 4.21 mmHg (95% CI − 4.66 to − 3.76), and RASI reduced MBPS by 6.90 mmHg (95% CI − 11.49 to − 2.31). No heterogeneity (I^2^ = 0%) was observed in the CCB subgroup, while high heterogeneity (I^2^ = 97%) was detected in the RASI subgroup. No differences in blood pressure reduction were observed with β-blockers (*P* = 0.67) and combined CCB and ARB (*P* = 0.52) in the evening versus morning administration.

The effects of intervention were also analyzed when MBPS was measured as categorical variable (Additional file 1: Fig. 1). No significance was detected in overall analysis. Due to the low number of trials analyzing MBPS as a categorical variable and different thresholds of MBPS, it might be insufficient to carry out a combined analysis.

#### Changes in SBP

**Fig. 3:**
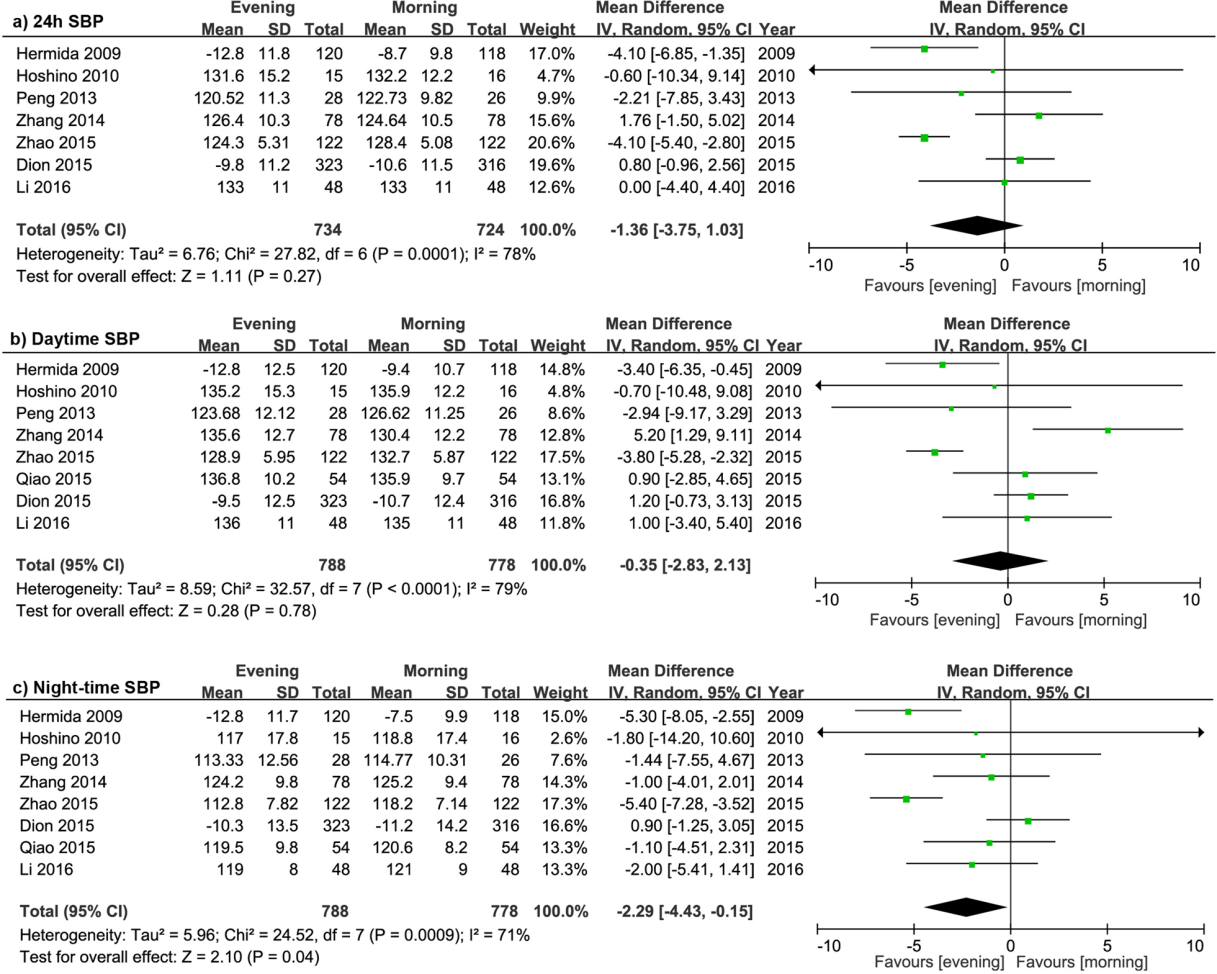
Forest plot: evening versus morning dosing regimen in 24 h SBP, daytime SBP and night-time SBP. SBP, systolic blood pressure; DBP, diastolic blood pressure

The data and results of SBP are presented in Fig. 3. The overall effects of evening administration showed no statistically significant reduction in 24-h SBP (*P* = 0.27) and daytime SBP (*P* = 0.78). However, evening regimen significantly decreased night-time SBP by 2.29 mmHg (95% CI − 4.43 to − 0.15), compared with morning administration. The subgroup analysis (Additional file 1: Table 4) showed that CCB evening regimen significantly reduced 24-h SBP, daytime SBP and night-time SBP by 4.1 mmHg (95% CI − 5.28 to − 2.92, I^2^ = 0%), 3.72 mmHg (95% CI − 5.04 to − 2.39, I^2^ = 0%) and by 5.37 mmHg (95% CI − 6.92 to − 3.82, I^2^ = 0%), respectively. There were no significant differences of SBP reduction were detected in RASI and CCB + ARB combined therapy.

#### Changes in DBP

**Fig. 4:**
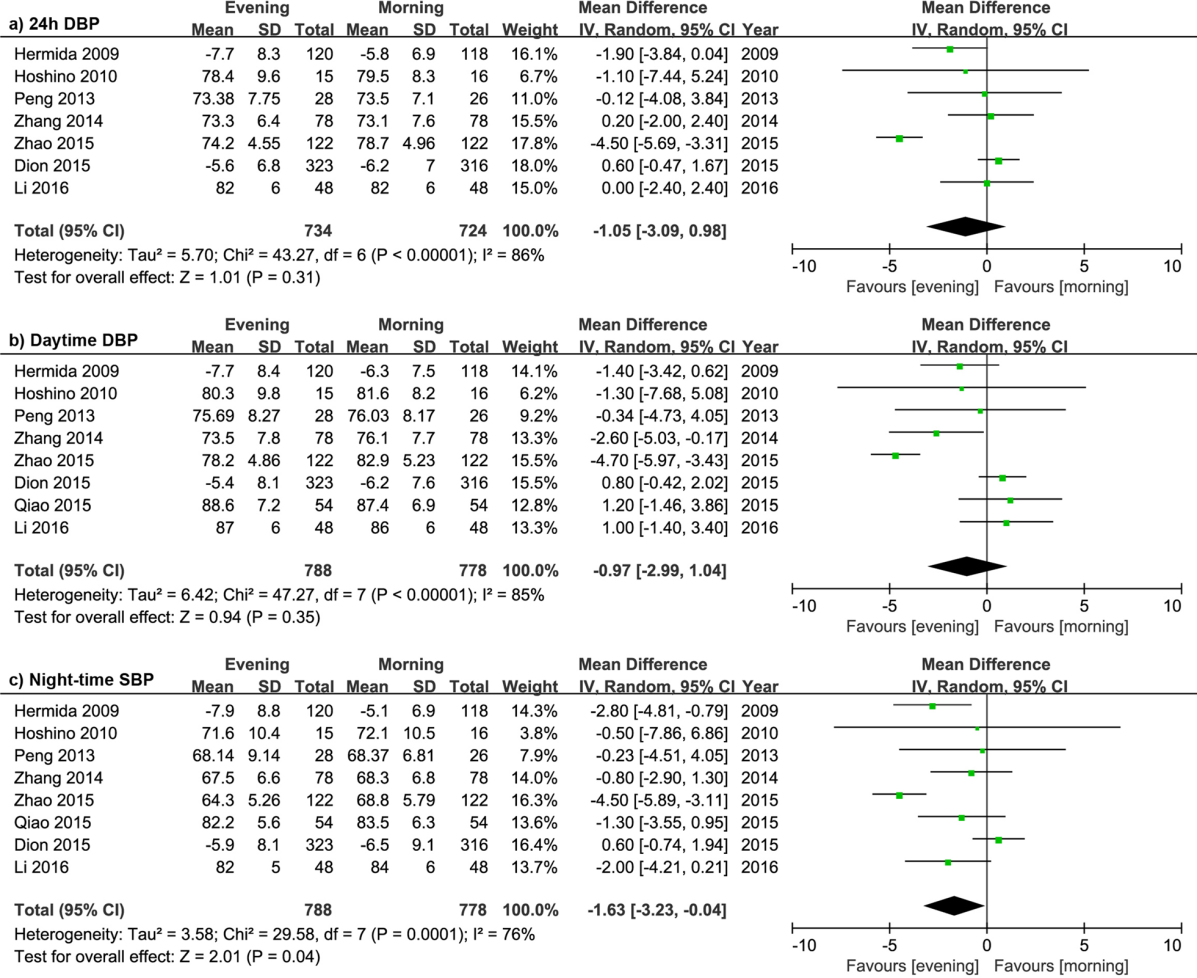
Forest plot: evening versus morning dosing regimen in 24 h DBP, daytime DBP and night-time DBP. DBP, diastolic blood pressure

The details of efficacy in DBP are shown in Fig. 4. The analysis of overall effects demonstrated that evening dosage regimen significantly reduced night-time DBP by 1.63 mmHg (*P* = 0.04) and no significant differences were observed in 24-h DBP (*P* = 0.31), daytime DBP (*P* = 0.35). The subgroup analysis (Additional file 1: Table 4) showed that the evening CCB was associated with significant 24-h DBP and night-time DBP reduction by 3.32 mmHg (95% CI − 5.85 to − 0.78) and 3.81 mmHg (95% CI − 5.45 to − 2.18), respectively; and no significant differences of DBP reduction were found in RASI or CCB + ARB combined therapy subgroups.

#### Changes in night BP dipping

6 studies with 3674 hypertensive subjects were included and analyzed. The result indicated that bedtime dosing remarkably enhanced nocturnal blood pressure decline by 3.23% (95% CI 5.37 to 1.10), compared with morning dosage regimen (Additional file 1: Fig. 2). And a high heterogeneity (I^2^ = 89%) was detected.

#### Adverse effects

The meta-analysis showed that the incidence of overall adverse effects (RR 0.65, 95% CI 0.30 to 1.41, I^2^ = 69%) and discontinuations due to adverse effects (RR 0.95, 95% CI 0.53 to 1.71, I^2^ = 0%) had no significant differences between evening and morning regimens (Additional file 1: Fig. 3 and Table 5). Among all the kinds of adverse effects reported, headache was the most frequently reported symptom, and a slightly significant increase of nausea was observed in the morning regimen (*P* = 0.05).

## Discussion

This systematic review and meta-analysis investigated the effects of evening and morning dosage of antihypertensive medications on MBPS and blood pressure control in patients with essential hypertension.

Morning administration of medication is commonly used in antihypertensive treatment in clinical practice. However, some recent clinical trials reported evening administration of the antihypertensive drug to be efficient in lowering blood pressure, improving organ functions, and preventing cardiovascular events [27–30]. This study indicated that the evening regimen of antihypertensive drugs exerted better effects on decreasing night-time blood pressure than morning therapy. Since studies showed that night-time blood pressure was significantly better than daytime blood pressure in predicting all-cause, cardiovascular and non-cardiovascular mortality, stroke and cardiovascular events [31, 32]. Besides, this analysis also demonstrated that bedtime administration significantly better enhanced night blood pressure dipping, relative to morning regimen, which was consistent with several previous studies. Increased nocturnal decline drives blood pressure towards more of a dipper pattern, thus improving blood pressure variability and lowering cardiovascular risk [33, 34]. Therefore, chronotherapy targeting night-time blood pressure control may favorably affect cardiovascular morbidity and mortality in hypertensive patients.

This study found that evening administration of blood pressure-lowering medication significantly improved MBPS, which has not been systematically reviewed before. The increase of MBPS was first associated with the incidence of stroke in 2003 [8]. A clinical trial with 519 hypertensive participants found that a 10 mm Hg raised in MBPS caused a 25% increased stroke incidence. Several but not all subsequent studies indicated that exaggerated MBPS had been related to increased risk for cardiovascular events and all-cause mortality. However, in recent years, the prognostic role of MBPS is controversial.

One possible reason is the distinct definitions and cut-off points of MBPS. There are four definitions commonly used (the sleep-trough surge, the prewaking surge, the rising blood pressure surge, and the morning-evening difference), and the thresholds are various. The thresholds of MBPS could be defined as top decile, quartiles of MBPS in participants, or determined according to hypertensive guidelines, which have not reached a consensus [35]. Among included studies, two articles analyzed MBPS as categorical variable with different predetermined threshold, 23.58 mmHg and 35 mmHg, respectively. Due to the limited number and different thresholds of studies, the heterogeneity between studies of categorical MBPS is very high. And a previous meta-analysis found that there was no significant association between MBPS and all-cause mortality or cardiovascular events when MBPS was analyzed as categorical variable [9]. Therefore, a single threshold dichotomizing MBPS as normal and exaggerated may not be powerful to define the significance of MBPS. Besides, in patients with non-dipper hypertension and nocturnal hypertension, which are described as a sleep-to-awake SBP ratio of less than 10%, and night-time SBP more than 120 mmHg and DBP more than 70 mmHg respectively [36], the MBPS may be low and not suitable for the evaluation of cardiovascular prognosis. This was also supported by the Jackson Heart Study, that no significant association between MBPS and the incidence of cardiovascular events was found in the black population with non-dipper and nocturnal hypertension [37]. Consequently, more research is needed to analyze MBPS’s definitions and reach a consensus on the target value of MBPS control. In patients with nocturnal dipping hypertension, a prognostic indicator needs to be developed to predict cardiovascular events and serve as a target of hypertension management.

On the other hand, ethnicity is a critical factor affecting morning blood pressure surge. The Ohasama Study [7] and JMS-ABPM Study [8] found significant relationship between exaggerated MBPS and stoke incidence in Japanese patients. Conversely, Bombelli et al. [38] reported that high MBPS was not associated with increased mortality and cardiovascular events in a white population. Furthermore, systematic review showed that the degree of sleep-trough surge was higher in Japanese than in European patients with hypertension [39]. The Jackson Heart Study also revealed that there was no clear evidence for the associations of sleep-trough MBPS, prewaking MBPS, and rising blood pressure surge with the incidence of cardiovascular events and all-cause mortality in black adults [37]. Discrepancies in the pathogenesis and manifestations of hypertension and related cardiovascular outcomes have been consistently reported among different races. Asians have more active sympathetic nerve activity during the morning period and higher incidence of stroke, which may account for the high degree and the prognostic role of MBPS [4]. Therefore, therapeutic strategies targeting MBPS control are of considerable significance in Asian population.

This meta-analysis discovered a prominent role of evening CCB in lowering blood pressure. The CCBs in included studies were nifedipine gastrointestinal therapeutic system (GITS) and amlodipine, which both belongs to the dihydropyridine calcium blocker family with long duration of action [40]. Amlodipine has been shown to be more effective for the MBPS control compared with valsartan in the VALUE trial [41]. Besides, previous evidence observed that the pharmacokinetic pharmacodynamics relationship of CCB is circadian rhythm-dependent that a night dosing of amlodipine displayed a longer half-life and higher peak plasma concentration compared to the morning dosage [42, 43]. This may also explain the remarkable effects of CCB in lowering night-time and MBPS when given at bedtime.

## Limitations

This study has some potential limitations. First, the searching results indicated that only ten studies were eligible for combined analysis, and six of them were conducted in China, which reflects the racial differences in the attention to MBPS. The lack of evidence also posed a limitation to the analysis of distinct definitions and thresholds of MBPS and sensitivity analysis according to methodological quality. Besides, the blood pressure-lowering efficacy alone is not adequate to judge the clinical significance of antihypertensive of chronotherapy. Thus long-term, larger scale and multi-racial clinical trials are needed to explore the correlation between MBPS reduction and the incidence of cardiovascular endpoint.

## Conclusions

MBPS has received considerable attention from clinicians. Our meta-analysis provided the evidence that evening administration of antihypertensive medication improves blood pressure variability by significantly reducing MBPS and increasing nocturnal blood pressure decline without increasing adverse effects.

## Supplementary Information

**Additional file 1**. Search strategy, description of MBPS and supplementary results of meta-analysis.

## Data Availability

All data generated or analyzed during this study are included in this article and additional information files.

## Abbreviations

ABPM: Ambulatory blood pressure monitoring
MBPS: Morning blood pressure surge
RCT: Randomized control trials
β-blockers: Adrenergic beta-antagonists
CCB: Calcium channel blockers
RASI: Renin-angiotensin system inhibitors
SBP: Systolic blood pressure
DBP: Diastolic blood pressure
SD: Standard deviation
RR: Relative risk ratios
GITS: Gastrointestinal therapeutic system
