# Supplementary material for: Chronotherapy for morning blood pressure surge in hypertensive patients: a systematic review and meta-analysis
Source: BMC Cardiovasc Disord. 2021 Jun 4;21:274. doi: 10.1186/s12872-021-02081-8 (PMC8176711; doi:10.1186/s12872-021-02081-8)

**Additional Appendix**

**Additional Table 1:** The search strategy used for Ovid Medline, Embase, Cochrane Library and SinoMed

**Additional Table 2:** Description of MBPS in included studies (definition and measurement of MBPS)

**Additional Table 3:** Risk of bias assessments table

**Additional Table 4:** Results of the subgroup analysis for different antihypertensive classes

**Additional Table 5:** Adverse effects reported during the trials

**Additional Figure 1:** Forest plot: evening versus morning dosing regimen in categorical MBPS

**Additional Figure 2:** Forest plot: evening versus morning dosing regimen in night blood pressure dipping

**Additional Figure 3:** Forest plot: overall adverse effects and adverse effects leading to discontinuation in evening and morning regimen

**Additional Table 1: The search strategy**

**Ovid Medline 1860-2020**

1 Hypertension/

2 blood pressure$.mp.

3 hypertens$.mp.

4 exp blood pressure/

5 1 or 2 or 3 or 4

6 exp Chronotherapy/

7 (chronopharm$ or chronomodulat$ or chronotherap$).mp.

8 6 or 7

9 (morning or day or am or diurnal$ or daytim$ or awak$).mp.

10 (evening or bedtim$ or night$ or nocturnal$ or pm).mp.

11 9 and 10

1. 8 or 11
2. (morning surge or morning blood pressure surge).ti,ab.
3. MBPS.mp.
4. 13 or 14
5. ((evening or night* or nocturnal) adj3 (dip* or difference or decline or decrease*)).tw.
6. ("night or day" or "day to night" or night?day or day?night).tw.
7. 16 and 17
8. 15 OR 18
9. 5 and 12 and 19
10. randomized controlled trial.pt.
11. controlled clinical trial.pt.
12. randomized.ab.
13. placebo.ab.
14. drug therapy.fs.
15. randomly.ab.
16. trial.ab.
17. groups.ab.
18. 21 or 22 or 23 or 24 or 25 or 26 or 27 or 28
19. (animals not (humans and animals)).sh.
20. 29 not 30
21. 19 and 31

**Embase 1966-2020**

#1 random* OR factorial* OR crossover* OR placebo* OR assign* OR allocat* OR volunteer* OR doubl* NEAR/5 blind* OR singl* NEAR/5blind*

#2 'crossover procedure'/exp

#3 'double-blind procedure'/exp

#4 'randomized controlled trial'/exp

#5 'single blind procedure'/exp

#6 #1 OR #2 OR #3 OR #4 OR #5

#7 'hypertension'/exp

#8 hypertens*

#9 'blood pressure'/exp

#10 #7 OR #8 OR #9

#11  'chronotherapy'/exp

#12 chronopharm* OR chronomodulat* OR chronotherap*

#13 morning OR day OR am OR diurnal* OR daytim* OR awak

#14 evening OR bedtim* OR night* OR nocturnal* OR pm

#15 #13 AND #14

#16 #11 OR #12 OR #15

#17 'morning surge'/exp OR 'morning blood pressure surge'/exp OR 'morning blood pressure surge'

#18 ((evening OR night* OR nocturnal) NEAR/3 (dip* OR difference OR decline OR decrease*)):ab

#19 #17 OR #18

#20 #6 AND #10 AND #16 AND #19

**The Cochrane Library database 2000-2020**

#1 (morning blood pressure surge):ti,ab,kw

#2 (MBPS):ti,ab,kw

#3 #1 OR #2

#4 (hypertension):ti,ab,kw

#5 MeSH descriptor: [Essential Hypertension] explode all trees

#6 #4 OR #5

#7 ("chronotherapy"):ti,ab,kw

#8 MeSH descriptor: [Drug Chronotherapy] explode all trees

#9 #7 OR #8

#10 (morning OR day OR am OR diurnal OR daytime OR awake):ti,ab,kw

#11 (evening OR bedtime OR night OR nocturnal OR pm):ti,ab,kw

#12 #10 AND #11

#13 #9 OR #12

#14 (night dipping OR night decline OR night fall OR evening dipping OR evening decline OR evening fall OR nocturnal dipping OR nocturnal decline OR nocturnal fall):ti,ab,kw

#15 #13 OR #14

#16 #3 AND #6 AND #15

**SinoMed**

12 (#11) AND (#9) AND (#9)

11 (#10) OR (#3)

10 (#2) OR (#1)

9 (#8) OR (#7) OR (#6) OR (#5)

8 (早晨 or 起床 or 凌晨 or 早间 or 上午) and (晚上 or 夜间 or 睡前)

7 (早上 or ⽩天 or 醒后 or 清晨) and (晚上 or 夜间 or 睡前)

6 "时间治疗学"[不加权:扩展]

5 "时间疗法"[不加权:扩展]

4 "高血压"[不加权:扩展]

3 "夜间血压下降率"[常用字段:智能]

2 血压晨峰现象

1 "晨峰血压"[常用字段:智能]

**Additional Table 2: Description of MBPS in included studies (definition and measurement of MBPS).**

| **Stud** | **Definition of MBPS** | **Method of BP**  **measurement** | **Measuring Device**  **(model)** | **Categorical**  **or continuous variable** | **Threshold** |
| --- | --- | --- | --- | --- | --- |
| Hermida et al. | sleep-trough surge | 24-h ABPM | SpaceLabs 90207 device | Continuous | None used |
| Hoshino et al | sleep-trough surge | 24-h ABPM | ABPM device (TM-2431) | Continuous | None used |
| Acelajado et al. | sleep-trough surge AND  Prewaking surge | 24-h ABPM | Oscar-2 device, SunTech Medical | Continuous | None used |
| Peng et al. | sleep-trough surge | 24-h ABPM | MGY-ABP l | Categorical | ≥35 mm Hg |
| Zhang et al. | sleep-trough surge | 24-h ABPM | MOBIL-O-GRAPH | Both | ≥23.58 mm Hg |
| Dion et al. | sleep-trough surge | 24-h ABPM | Not stated | Continuous | None used |
| Lai et al | sleep-trough surge | 24-h ABPM | MOBIL-O-GRAPH | Continuous | ≥23 mm Hg |
| Qiao et al. | sleep-trough surge | 24-h ABPM | MEIGAOYIMGY -ABP1 | Continuous | ≥23.58 mm Hg |
| Zhao et al. | sleep-trough surge | 24-h ABPM | Spacelabs.90217 | Continuous | None used |
| Li et al | sleep-trough surge | 24-h ABPM | WelchAllyn-6100 | Continuous | ≥35 mm Hg |

Abbreviation: MBPS, morning blood pressure surge; ABPM, ambulatory blood pressure measurement.

**Additional Table 3: Risk of bias assessments table.**

| **Studies** | Random Sequence Generation  **(selection bias)** | Allocation Concealment  **(selection bias)** | Blinding Participants and Personnel  **(performance bias)** | Blinding of outcome Assessment  **(detection bias)** | Incomplete Outcome Data  **(attrition bias)** | Selective Reporting  **(reporting bias)** | Other bias |
| --- | --- | --- | --- | --- | --- | --- | --- |
| **Hermida 2009** | low | low | low | low | low | low | low |
| **Hoshino 2010** | low | low | low | low | low | low | unclear **^a^** |
| **Acelajado 2012** | low | low | low | low | low | unclear | high **^b^** |
| **Peng 2013** | low | low | low | low | low | unclear | unclear **^a^** |
| **Zhang 2014** | low | low | low | low | low | unclear | unclear **^a^** |
| **Dion 2015** | low | low | low | low | low | low | low |
| **Lai 2015** | low | low | low | low | low | low | unclear **^a^** |
| **Qiao 2015** | low | low | low | low | low | unclear | high **^c^** |
| **Zhao 2015** | low | low | low | low | low | unclear | unclear **^a^** |
| **Li 2016** | low | low | low | low | low | unclear | unclear **^a^** |

NOTE: The assessment of risk of bias were according to Cochrane risk of bias assessments tool.

Abbreviation: low, low risk of bias; unclear, unclear risk of bias; high, high risk of bias.

**^a^** Insufficient information to assess whether an important risk of bias exist.

**^b^** As a cross-over RCT, only the first-period data are available.

**^c^** Baseline imbalance. No information about patients' baseline BP and mALB. In addition to intervention, patients haven't stopped their "conventional treatments", which may have influence on outcomes.

**Additional Table 4: Results of the subgroup analysis for different antihypertensive classes.**

|  | **No. of studies** | **No. of participants** | **Mean**  **difference**  **(95% CI)** | **Test for overall**  **effect Z (P)** | **I^2^% (P)** | **Test for subgroup**  **differences** |
| --- | --- | --- | --- | --- | --- | --- |
| **24-h SBP** | 7 | 1458 | -1.39 (-3.89 to 1.11) | Z =1.09 (P =0.28) | 82% (<0.0001) | (Chi^2^ =24.02; P ＜0.00001 ) |
| CCB | 2 | 482 | -4.10 (-5.28 to -2.92) | Z =6.82 (P < 0.00001)*** | 0% (P =1.00) |  |
| RASI | 2 | 735 | 0.69 (-0.94 to 2.32) | Z =0.83 (P =0.41) | 0% (P =0.74) |  |
| Combined therapy | 3 | 241 | 0.66 (-2.06 to 3.37) | Z =0.47 (P =0.63) | 0% (P =0.47) |  |
| **24-h DBP** | 7 | 1458 | -1.05 (-3.19 to 1.10) | Z =0.96 (P =0.34) | 88% (<0.00001) | (Chi^2^ =7.58; P =0.02) |
| CCB | 2 | 482 | -3.32 (-5.85 to -0.78) | Z =2.56 (P =0.01)** | 80% (P =0.03) |  |
| RASI | 2 | 735 | -0.50 (-0.48 to 1.48) | Z =1.00 (P =0.32) | 0% (P =0.65) |  |
| Combined therapy | 3 | 241 | 0.02 (-1.82 to 1.86) | Z =0.02 (P =0.98) | 0% (P =0.93) |  |
| **Daytime SBP** | 8 | 1566 | -0.33 (-2.93 to 2.27) | Z =0.5 (P =0.80) | 82% (<0.0001) | (Chi^2^ =21.48; P ＜0.0001 ) |
| CCB | 2 | 482 | -3.72 (-5.04 to -2.39) | Z =5.50 (P < 0.00001)*** | 0% (P =0.81) |  |
| RASI | 3 | 843 | 1.12 (-0.48 to 2.72) | Z =1.37 (P =0.17) | 0% (P =0.99) |  |
| Combined therapy | 3 | 241 | 1.16 (-4.66 to 6.98) | Z =0.39 (P =0.70) | 61% (P =0.08) |  |
| **Daytime DBP** | 8 | 1566 | -0.95 (-3.06 to 1.17) | Z =0.88 (P =0.38) | 87% (<0.00001) | (Chi^2^ =10.27; P =0.006 ) |
| CCB | 2 | 482 | -3.15 (-6.38 to 0.08) | Z =1.91 (P =0.06) | 86% (P =0.06) |  |
| RASI | 3 | 843 | 0.89 (-0.11 to 1.90) | Z =0.88 (P =0.38) | 0% (P =0.96) |  |
| Combined therapy | 3 | 241 | -1.99 (-4.01 to 0.03) | Z =1.93 (P =0.05)** | 0% (P =0.66) |  |
| **Night-time SBP** | 8 | 1566 | -2.30 (-4.52 to -0.08) | Z =2.03 (P =0.04)** | 76% (P =0.0004) | (Chi^2^ =19.74; P ＜0.0001 ) |
| CCB | 2 | 482 | -5.37 (-6.92 to -3.82) | Z =6.78 (P < 0.00001)*** | 0% (P =0.95) |  |
| RASI | 3 | 843 | -0.28 (-2.06 to 1.50) | Z =0.31 (P =0.76) | 15% (P =0.31) |  |
| Combined therapy | 3 | 241 | -1.12 (-3.76 to 1.52) | Z =0.83 (P =0.41) | 0% (P =0.99) |  |
| **Night-time DBP** | 8 | 1566 | -1.68 (-3.34 to -0.02) | Z =1.98 (P =0.05)** | 80% (<0.0001) | (Chi^2^ =8.74; P =0.01 ) |
| CCB | 2 | 482 | -3.81 (-5.45 to -2.18) | Z =4.57 (P < 0.00001)*** | 46% (P =0.17) |  |
| RASI | 3 | 843 | -0.70 (-2.40 to 1.00) | Z =0.81 (P =0.42) | 58% (P =0.09) |  |
| Combined therapy | 3 | 241 | -0.68 (-2.51 to 1.15) | Z =0.73 (P =0.47) | 0% (P =0.97) |  |

Abbreviations: SBP, systolic blood pressure; DBP, diastolic blood pressure; CCB, calcium channel blockers; RASI, renin-angiotensin system inhibitors.

P values for trend, **: p≤0.05; ***: p ≤0.001.

**Additional Table 5:** **Adverse effects reported during the trials.**

|  | **Hermida 2009** | | **Zhao 2015** | | **Dion 2015** | | **RR (95%CI)** | **P value** |
| --- | --- | --- | --- | --- | --- | --- | --- | --- |
|  | Evening  (n=120) | Morning  (n=118) | Evening  (n=122) | Morning  (n=122) | Evening  (n=323) | Morning  (n=316) |  |  |
| Headache | 1  (0.83%) | 1  (0.85%) | 2  (1.64%) | 3  (2.46%) | 12  (3.72%) | 13  (4.11%) | 0.87 (0.44, 1.72) | 0.68 |
| Nasopharyngitis | NA | NA | NA | NA | 8  (2.48%) | 9  (2.85%) | 0.87 (0.34, 2.23) | 0.77 |
| Edema | 1  (0.83%) | 13  (11%) | 3  (2.46%) | 3  (2.46%) | NA | NA | 0.29 (0.02, 4.06) | 0.86 |
| Bronchitis | NA | NA | NA | NA | 15  (4.64%) | 8  (2.53%) | 1.83 (0.79, 4.27) | 0.16 |
| Nicturia | 1  (0.83%) | 0 | NA | NA | NA | NA | 2.95 (0.12, 71.70) | 0.61 |
| Cough | NA | NA | NA | NA | 12  (3.72%) | 8  (2.53%) | 1.47 (0.61, 3.54) | 0.39 |
| Nausea | NA | NA | NA | NA | 1  (0.31%) | 8  (2.53%) | 0.12 (0.02, 0.97) ** | 0.05 |
| Vertigo | NA | NA | NA | NA | 6  (1.86%) | 6  (1.9%) | 0.98 (0.32, 3.00) | 0.97 |
| Constipation | NA | NA | 2  (1.64%) | 3  (2.46%) | NA | NA | 0.67 (0.11, 3.92) | 0.65 |
| Palpitations | 0 | 1  (0.85%) | NA | NA | NA | NA | 0.33 (0.01, 7.97) | 0.49 |
| Upper abdominal pain | NA | NA | NA | NA | 3  (0.93%) | 5  (1.58%) | 0.59 (0.14, 2.44) | 0.46 |
| Diarrhoea | NA | NA | NA | NA | 4 | 4 | 0.98 (0.25, 3.88) | 0.98 |
| Back pain | NA | NA | NA | NA | 9 | 3 | 2.93 (0.80, 10.74) | 0.10 |
| Skin rash | 1  (0.83%) | 2  (1.69%) | NA | NA | NA | NA | 0.49 (0.05, 5.35) | 0.56 |

NOTE: Data are presented as n (%); n, number of patients with at least one event.

P values for trend, **: p≤0.05.

Abbreviation: RR, risk ratios; NA: Not available.

**Additional Figure 1: Forest plot: evening versus morning dosing regimen in categorical MBPS**


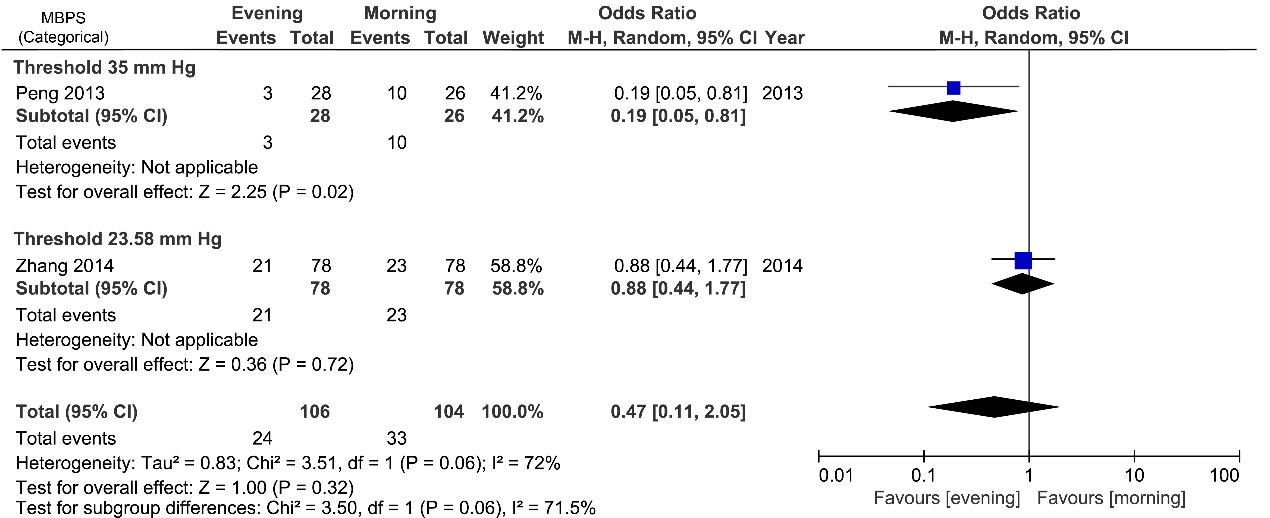


**Additional Figure 2: Forest plot: evening versus morning dosing regimen in night blood pressure dipping**


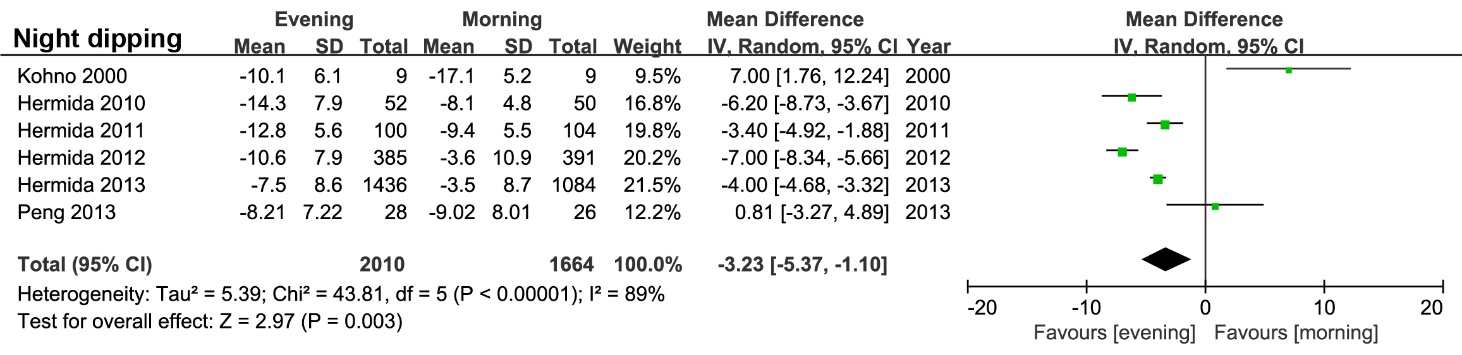


**Additional Figure 3: Forest plot: overall adverse effects and adverse effects leading to discontinuation in evening and morning regimen**


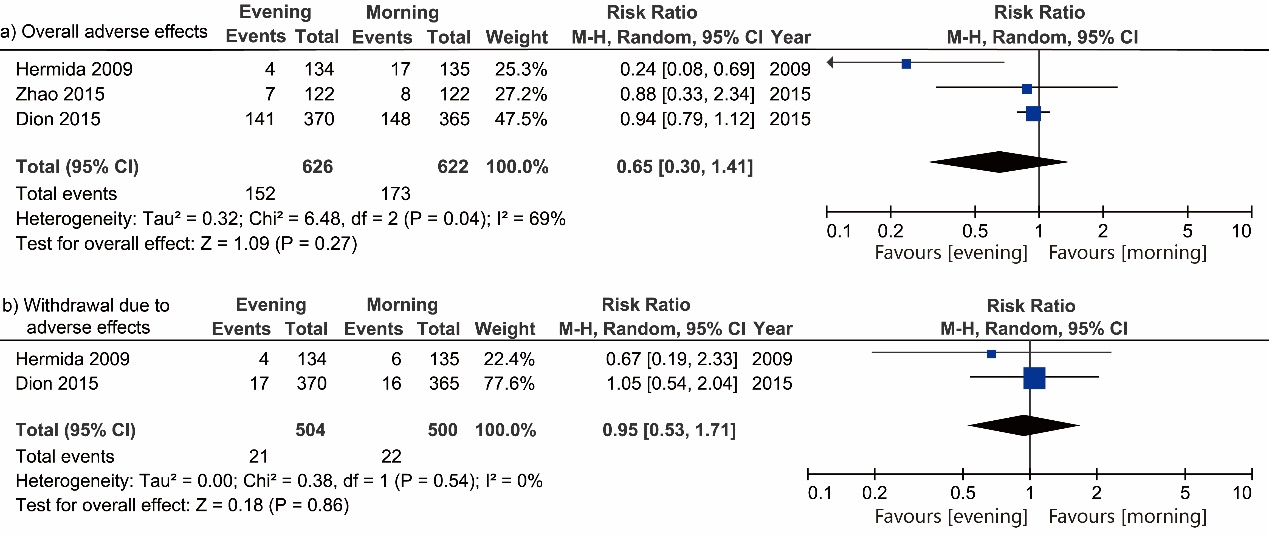

Supplement: Supplementary file 1 — Additional file 1. Search strategy, description of MBPS and supplementary results of meta-analysis. [file 12872_2021_2081_MOESM1_ESM.docx]
